# Targeted therapy for hepatocellular carcinoma: novel agents on the horizon

**DOI:** 10.18632/oncotarget.466

**Published:** 2012-03-31

**Authors:** Melchiorre Cervello, James A. McCubrey, Antonella Cusimano, Nadia Lampiasi, Antonina Azzolina, Giuseppe Montalto

**Affiliations:** ^1^ Institute of Biomedicine and Molecular Immunology, “Alberto Monroy” National Research Council (C.N.R.) Via Ugo La Malfa 153, 90146 Palermo, Italy; ^2^ Department of Microbiology and Immunology, Brody School of Medicine at East Carolina University, 600 Moye Blvd, Greenville NC 27858, USA; ^3^ Department of Internal Medicine and Specialties, University of Palermo, Via del Vespro 143, 90127 Palermo, Italy

**Keywords:** HCC, targeted therapy, VEGF, Ras/Raf/MEK/ERK, PI3K/Akt/PTEN/mTOR, signal transduction inhibitors, cancer

## Abstract

Hepatocellular carcinoma (HCC) is the most common liver cancer, accounting for 90% of primary liver cancers. In the last decade it has become one of the most frequently occurring tumors worldwide and is also considered to be the most lethal of the cancer systems, accounting for approximately one third of all malignancies.

Although the clinical diagnosis and management of early-stage HCC has improved significantly, HCC prognosis is still extremely poor. Furthermore, advanced HCC is a highly aggressive tumor with a poor or no response to common therapies. Therefore, new effective and well-tolerated therapy strategies are urgently needed.

Targeted therapies have entered the field of anti-neoplastic treatment and are being used on their own or in combination with conventional chemotherapy drugs. Molecular-targeted therapy holds great promise in the treatment of HCC. A new therapeutic opportunity for advanced HCC is the use of sorafenib (Nexavar). On the basis of the recent large randomized phase III study, the Sorafenib HCC Assessment Randomized Protocol (SHARP), sorafenib has been approved by the FDA for the treatment of advanced HCC. Sorafenib showed to be able to significantly increase survival in patients with advanced HCC, establishing a new standard of care. Despite this promising breakthrough, patients with HCC still have a dismal prognosis, as it is currently the major cause of death in cirrhotic patients. Nevertheless, the successful results of the SHARP trial underscore the need for a comprehensive understanding of the molecular pathogenesis of this devastating disease.

In this review we summarize the most important studies on the signaling pathways implicated in the pathogenesis of HCC, as well as the newest emerging drugs and their potential use in HCC management.

## INTRODUCTION

Hepatocellular carcinoma (HCC) is the most common liver cancer, accounting for 90% of primary liver cancers. In the last decade it has become one of the most frequently occurring tumors worldwide and is also considered to be the most lethal of the cancer systems, accounting for approximately one third of all malignancies [1, 2]. Distribution, however, is not homogeneous around the world, as important differences have been noted between countries, with most cases occurring in Eastern Asia and sub-Saharan Africa, while low rate areas are in North America, northern Europe and Australia [3, 4]. Changes in liver cancer incidence are beginning to be reported, namely a certain degree of reduction in the high-rate areas, particularly in China, thanks to the implementation of universal hepatitis B virus vaccination and limitation to aflatoxin B1 exposure [5, 6], while increasing incidences are being reported in low-rate areas, particularly in the United Kingdom and Australia [7].

By far the most frequent risk factor for HCC is liver cirrhosis (LC), this underlying disease being present in a variable proportion of cases, reaching a 90% rate in western countries [8]. The main etiological agents of LC are the hepatitis B (HBV) and hepatitis C (HCV) viruses, which together account for three quarters of all HCC cases worldwide. The diffusion of these viruses in the world reflects regional differences in the quantitative and qualitative (etiological) pattern of HCC. Other risk factors include aflatoxin B1 intake, alcohol consumption, non-alcoholic fatty liver disease (NAFLD) and some hereditary diseases, including hereditary hemochromatosis [9]. In the last few years a great body of evidence has been reported about the possibility that some severe forms of NAFLD may progress to HCC. NAFLD is usually part of the metabolic syndrome, found namely in patients with diabetes mellitus, hypertension, dyslipidemia, obesity and insulin resistance, which is becoming very frequent in western populations, due to their life style (sedentariness) and diet. It has also been called into question in many cases of HCC of “cryptogenetic” origin [10-12]. In particular, several studies suggest that obese patients are also at increased risk for several types of cancer, including HCC. Recently, a meta-analysis found that the relative risks (RR) for liver cancer were higher in obese (Body Mass Index, BMI ⩾ 30) than in overweight subjects (BMI = 25–30) [13].

HCC predominantly affects males, with a male to female ratio averaging 2:1 and 4:1 [9], although after the menopause no significant differences have been reported between the sexes [14]. For this reason sex hormones have been thought to play a possible role in neoplastic degeneration and various therapeutic evaluations based on anti-androgen or anti-estrogen agents have been performed, albeit with disappointing results [15].

We can therefore state that the pathogenesis of HCC is very complex and not completely clear. As in most cancers, HCC pathogenesis is a multistep process, involving sequential events such as chronic inflammation, hyperplasia and dysplasia and ultimately malignant transformation. It is a very long process, which usually takes even up to 30 years and during these years there are a number of epigenetic and genetic alterations, ultimately leading to an alteration in the molecular pathways. Several results indicate that there is no dominant pathway specifically altered in HCC [16]. Indeed, there are several subclasses of tumors presenting distinct molecular aberrations responsible for cell proliferation and survival, while other alterations present in almost all tumors involve limitless replicative potential, neoangiogenesis, and insensitivity to antigrowth signals and checkpoint disruption [16]. Recent discoveries in the complex networks involved in HCC proliferation, progression and survival have created many opportunities for targeted drugs and new therapeutic approaches to this disease. These new targets include signal transduction pathways, oncogenes and growth factors and their receptors.

In this review we will focus on the most frequently dysregulated signaling pathways implicated in the pathogenesis of HCC, as well as the newest emerging drugs and their potential use in the management of HCC.

## SIGNALING PATHWAYS

**Figure 1:**
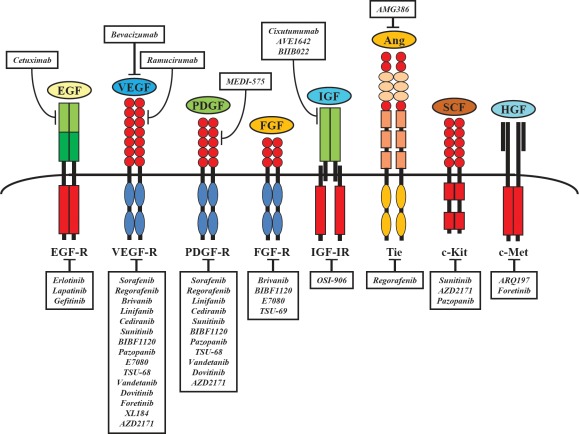
Relevant receptors and corresponding molecular targeted agents currently tested in preclinical and clinical HCC trials

**Figure 2:**
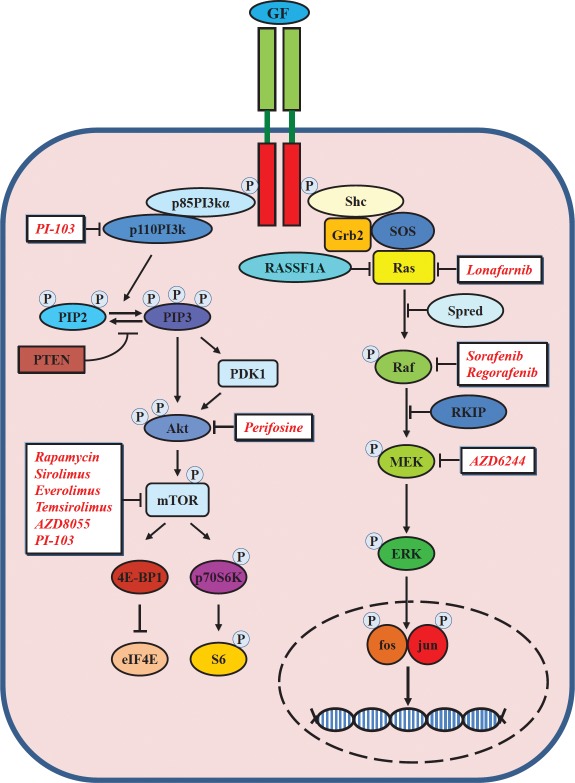
Schematic overview of PI3K/PTEN/Akt/mTOR and Ras/Raf/MEK/ERK signaling pathways stimulated after binding of a growth factor (GF) to a receptor tyrosine kinase

**Figure 3:**
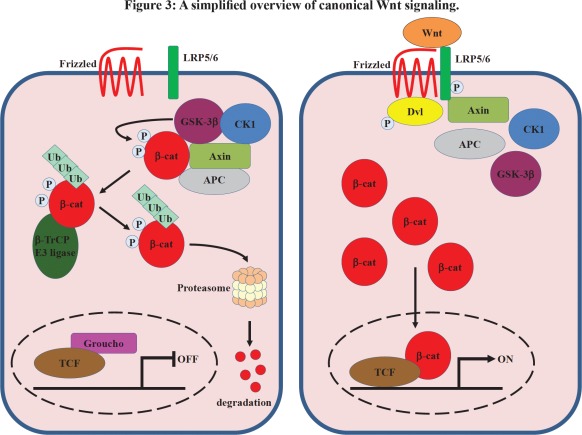
A simplified overview of canonical Wnt signaling

The key signal transduction pathways that have been implicated in the pathogenesis of HCC include those mediated by epidermal growth factor (EGF)/EGF receptor (EGFR), vascular endothelial growth factor (VEGF)/VEGF receptor (VEGFR), platelet-derived growth factor (PDGF)/PDGF receptor (PDGFR), insulin-like growth factor (IGF)/IGF receptor (IGFR), and the Ras/Raf/mitogen-extracellular activated protein kinase kinase (MEK)/ extracellular signal-regulated kinase (ERK), Wnt/β-catenin, and phosphatidylinositol-3-kinase (PI3K)/phosphatase and tensin homologue deleted on chromosome ten (PTEN)/Akt/mammalian target of rapamycin (mTOR) signaling pathways (Figures 1-3). Further attention is required to determine the relevance and therapeutic potential of other pathways involved in liver carcinogenesis, such as the interleukin 6 (IL-6), signal transducer and activator of transcription (STAT) and Hedgehog signaling pathways.

Activation of these pathways will eventually lead to resistance to apoptosis, cell proliferation, the stimulation of angiogenesis, invasiveness and metastasis. In the past decade there has been significant breakthroughs in the discovery of interacting pathway components and insights into how mutations of these components can lead to aberrant signaling, uncontrolled proliferation and even sensitivity/resistance to targeted therapy [17-19]. Research has resulted in to the development of inhibitors that specifically target critical elements of these pathways as well as the concept that mutations at one signaling molecule in the pathways (*e.g*., EGFR, Ras, B-Raf) may prevent sensitivity to an inhibitor targeting a downstream component (e.g., Raf, MEK or PI3K) [20-25]. These studies indicate that the mutational status of key genes in the pathway (*e.g.,* Ras, B-Raf) will have to be determined in cancer patients before applications of targeted therapy [17]. While sensitivity to EGFR inhibitors in non small cell lung carcinomas (NSCLC) is often due to mutations (L858R) or small deletions in exon 19 in the kinase domain, initial sensitivity to EGFR inhibitors may be lost due to subsequent mutations in the kinase domain. Other mutations in the kinase domain of EGFR prevent the induction of pro-apoptotic Bim in response to EGFR inhibitors. In some cases of NSCLC which have become resistant to EGFR inhibitors, they over express the c-Met proto-oncogene. Finally K-Ras mutations confer resistance to EGFR inhibitors (erlotinib, gefitinib or antibodies such as cetuximab). In some cases resistance to either Raf/MEK or PI3K may occur as some upstream mutations (*e.g*., EGFR mutations) activate both Raf/MEK/ERK and PI3K/PTEN/Akt/mTOR signaling pathways. Treatment of cells with Ras mutations with certain mutant allele selective B-Raf inhibitors can result in Raf-1 activation. Dominant negative B-Raf mutations can still bind and activate Raf-1 if the cell has a mutant Ras allele. Finally some B-Raf inhibitor resistant cells overexpress various critical cell cycle regulatory molecules such as cyclin D. The various mechanisms of inhibitor resistance involving other components in these pathways are explained in more detail in McCubrey et al. [17]. Many recent studies are directed at increasing cancer patient survival by targeting these and other pathways in cancer cells.

Illustrations of the most important receptors and intracellular molecular signaling pathways, as well as sites of intervention with small molecule inhibitors and monoclonal antibodies are presented in Figures 1-2. Certain molecular-targeted agents are actually promiscuous (*e.g.* sorafenib, regorafenib, sunitinib, brivanib and others), *i.e.* they simultaneously target more than one molecule and this multiple targeting could enhance their therapeutic efficacy, while others act on a single target (*e.g.* AZD6244, AZD8055, everolimus and others) (Figure 1).

## EGF/EGFR PATHWAY

The EGFR (also known as ErbB1 and Her1) belongs to the ERB family of receptor tyrosine kinases (RTKs), which includes ErbB2 (also known as Her2), ErbB3 (also known as Her3) and ErbB4 (also known as Her4). The members are all endowed with tyrosine kinase (TK) activity, with the exception of ErbB3. All members share a common structure, showing an extracellular ligand-binding domain, a transmembrane domain and an intracellular domain where the tyrosine kinase activity resides. EGFR forms homo- or heterodimers upon ligand binding. Dimerization results in auto-phosphorylation of EGFR with the subsequent activation of a number of downstream signaling pathways, including the PI3K/Akt/mTOR and the Ras/Raf/MEK/ERK pathways (Figure 2). With the exception of ErbB2, which has no ligand, all the other members can bind a family of growth factors. Ligands for EGFR are EGF, TGF-α, epigenin (EPG), amphiregulin (AREG), heparin-binding-EGF (HB-EGF), epirugulin (EREG) and β-cellulin (BTC) and the last three ligands are also able to bind to ErbB4/Her4. The neuregulin (NRG) ligands NRG-1 and NGR-2 bind to both ErbB3/Her3 and ErbB4/Her4, whereas NGR-3 and NGR-4 only recognize ErbB4/Her4.

The receptor most studied in HCC is EGFR/ErbB1. The rationale for targeting the EGFR pathway comes from the following observations: there is a high frequency of EGFR overexpression in HCC [26-29], and this overexpression has been associated with late-stage disease, increased cell proliferation and degree of tumor differentiation [28-30]. In addition, activation of the EGFR pathway is a prognostic predictor of survival in patients with HCC [31]. Therefore, EGFR represents a good potential molecular target for the biological therapy of HCC.

The importance of EGF/EGFR signaling in the development of HCC has been confirmed in two recent studies showing that cirrhotic patients with high levels of serum and tissue EGF have a higher adjusted risk of developing HCC compared to cirrhotic patients with EGF levels comparable to healthy subjects [32, 33]. High levels of EGF are due to the presence of a single-nucleotide polymorphism in the *EGF* gene, involving *A* to *G* transition at position 61 in the 5′ untranslated region of the *EGF* gene (SNP rs4444903). The transcript of patients with SNP exhibited more than a 2-fold longer half-life than those from the *wt* allele and serum EGF levels were 1.8-fold higher in *G/G* patients than *A/A* patients, while liver EGF levels were 2.4-fold higher in *G/G* patients than in *A/A* patients. Whether higher EGF levels are associated with a greater risk of developing cirrhosis and a shorter time taken to develop cirrhosis were aspects not addressed by this study. However, the observation that the severity of cirrhosis did not differ between *A/A*, *A/G*, and *G/G* patients argues against this possibility [32].

## RAS/RAF/MEK/ERK PATHWAY

The Ras/Raf/MEK/ERK pathway, also known as the MAPK (mitogen-activated protein kinase) pathway, is a signaling pathway consisting of a kinase cascade regulated by phosphorylation and de-phosphorylation by specific kinases and phosphatases as well as GTP/GDP exchange proteins, adaptor proteins and scaffolding proteins (Figure 2). In response to a variety of cellular stimuli, including growth factor-mediated activation of receptor tyrosine kinases (RTKs), Ras assumes an activated GTP-bound state, leading to recruitment of Raf from the cytosol to the cell membrane, where it becomes activated, likely via a Src-family tyrosine kinase [20, 21, 34-36]. Activated Raf causes the phosphorylation and activation of MAP kinase extracellular signal-regulated kinases 1 and 2 (MEK1/MEK2), which in turn phosphorylate and activate extracellular signal-regulated kinases 1 and 2 (ERK1/ERK2) at specific Thr and Tyr residues [37]. Activated ERK can translocate into the nucleus and phosphorylate additional transcription factors, such as Elk-1, CREB, Fos and globin transcription factor 1 (Gata-1) as well as others, which bind promoters of many genes, including growth factor and cytokine genes, which are important in promoting growth and preventing the apoptosis of multiple cell types [38-40].

Deregulation of the Ras/Raf/MEK/ERK pathway plays a key role in the pathogenesis of several human cancers [17, 41, 42], including HCC [21, 43-48]. Although mutations of Ras and Raf occur infrequently in HCC, a recent study demonstrated that activation of the Ras pathway was observed in 100% of HCC specimens analyzed when compared with non-neoplastic surrounding tissue and normal livers. This increased expression of Ras coincided with the decreased expression of genes which serve to inhibit Ras expression, namely the Ras-association domain family 1A (RASSF1A) and the novel Ras effector 1A (NORE1A). These genes may be suppressed due to aberrant methylation of their promoters [49]. In addition, activation of the Ras/Raf/MEK/ERK pathway in HCC may be due to the down-regulation of Ras inhibitors Sprouty and Sprouty-related protein with Ena/vasodilator-stimulated phosphoprotein homology-1 domain (Spred-1) and Spred-2 [50, 51]. It has been shown that the expression of Spred-1 and -2 in human HCC tissues is frequently lower than in the adjacent non-tumor tissue and inversely correlates with the incidence of tumor invasion and metastasis [51]. Moreover, forced expression of Spred inhibited HCC cell proliferation both *in vitro* and *in vivo*, which was associated with reduced ERK activation, suggesting that Spred could be not only a novel prognostic factor but also a new therapeutic target for human HCC [51]. Recently, studies have also shown that down-regulation of Raf kinase inhibitor protein (RKIP) expression is a major factor in the activation of the ERK/MAPK pathway during human liver carcinogenesis [52, 53].

Deregulation of the ERK pathway has clinical importance in HCC. Activation of the ERK signaling pathway predicts poor prognosis in hepatocellular carcinoma [54]. The important role of ERK signaling has also been suggested for HCC progression in obese patients. A possible explanation for an associated risk for obesity and HCC comes from the study of Saxena et al., which for the first time demonstrated that leptin, a key molecule involved in the regulation of energy balance and body weight control, promotes HCC growth and invasiveness through activation of ERK signaling [55].

Other well known risk factors for HCC such as HBV and HCV infection also seem to utilize the Raf/MEK/ERK pathway for the control of hepatocyte survival and viral replication [56, 57]. HBx, one of the four proteins encoded by the HBV genome, has been reported to be involved in liver carcinogenesis, with HBx expression activating the Ras, Raf, MAP kinase signaling cascade [56, 58-60]. Among the HCV components, the core protein has been reported to activate the Ras/Raf/MEK/ERK pathway and thereby might contribute to HCC carcinogenesis [57, 61, 62]. Therefore, these studies suggested the possible use of the Raf/MEK/ERK pathway as a target in therapeutic approaches for the treatment of HCC resulting from HBV and HCV infection. Taken together, these data suggest that the Raf/MEK/ERK pathway may represent an important therapeutic target for the treatment of HCC in patients with differing etiologies that lead to the development of this aggressive tumor.

Activation of Ras/Raf/MEK/ERK signaling in HCC may result from up-regulation of IGF [63], aberrant upstream EGFR signaling [64] and other receptor signaling (*i.e.* VEGFR and PDGFR). An effective blockade of the Ras/Raf/MEK/ERK pathway can be achieved using small molecules, such as lonafarnib, sorafenib, regorafenib, AZD6244 and others (Figures 1 and 2). Drugs inhibiting components of the Ras/Raf/MEK/ERK pathway, with the exception of sorafenib, are still in the pre-clinical phase or in phase I/II clinical trials for HCC therapy (Table 1).

**Table I:** Trials of molecular targeted agents in HCC

Agent as monotherapy	Target	Design	Clinicaltrials.gov Identifier
Sorafenib (Nexavar, BAY43-9006; *Bayer*)	BRAF, VEGFR-2, VEGFR-3, PDGFR-b, c-KIT, Flt3	Registered	
Regofarenib (fluoro-sorafenib, BAY73-4506; *Bayer*)	BRAF, VEGFR-2, VEGFR-3, PDGFR-b, c-KIT, Flt3, Tie2	Phase I/II	NCT01117623; NCT01003015
Sunitinib (Sutent, SU11248; *Pfizer*)	VEGFR-1 VEGFR-2, PDGFR-α, PDGFR-b, c-KIT, Flt3, RET, CSF-1R	Phase III	NCT00699374
Brivanib (BMS-582664; *Bristol-Meyers Squibb)*	VEGFR- 2, VEGFR-3, FGFR-2, FGFR-3	Phase III	NCT00858871; NCT00825955
Linifanib (ABT-869; *Abbott*)	VEGFR-2, PDGFR-b, CSF-1R	Phase II	NCT00517920; NCT01009593
Pazopanib (GW786034, Votrient; *GalxoSmithKline*)	VEGFR-1, VEGFR-2, VEGFR-3, PDGFR-α, PDGFR-b, c-KIT	Phase I	NCT00370513
TSU-68 (SU6668; *Taiho*)	VEGFR-2, PDGFR-β, FGFR-1	Phase I/II	NCT00784290
Foretinib (XL880, GSK1363089; *GlaxoSmithKline*)	VEGFR-2; c-MET	Phase I/II	NCT00920192
E7080 (*Eisai*)	VEGFR-1, VEGFR-2, VEGFR-3	Phase I/II	NCT00946153
BIBF 1120 (Vargatef; *Boeringer Ingelheim*)	VEGFR-2, PDGFR-b, FGFR	Phase II	NCT00987935
XL184 (BMS907351; *Bristol-Meyers Squibb*)	VEGFR-2; c-MET	Phase II	NCT00940225
Dovitinib (TKI258; *Novartis*)	VEGFR-1, VEGFR-2, VEGFR-3, PDGFR-b, FGFR-3, Flt3, c-KIT, CSF-1R	Phase II	NCT01232296
Cediranib (Recentin, AZD2171; *AstraZeneca)*	VEGFR-2	Phase II	NCT00427973; NCT00238394
Vandetanib (Zactima, ZD6474; *AstraZeneca)*	VEGFR, RET, EGFR	Phase I/II	NCT00496509; NCT00508001
Foretinib (XL880, GSK1363089; *GlaxoSmithKline*)	VEGFR-2, c-Met	Phase I	NCT00920192
Ramucirumab (IMC-1121B; *ImClone Systems Inc*)	VEGFR-2	Phase II/III	NCT00627042; NCT01140347
Bevacizumab (Avastin; *Genetech/Roche*)	VEGF	Phase II	NCT00162669
Erlotinib (Tarceva, OSI774; *Genetech*)	EGFR	Phase I/II	NCT00047346; NCT00047333
Lapatinib (Tyverb, GW572016; *GlaxoSmithKline*)	EGFR, HER2/neu	Phase II	NCT00107536; NCT00101036
Gefitinb (Iressa, ZD1839; *AtraZeneca*)	EGFR	Pahse II	NCT00071994; NCT00282100
Cetuximab (Erbitux, IMC-C225; *Bristol-Meyers Squibb, Merck Serono*)	EGFR	Phase II	NCT00142428
OSI-906 (*OSI Pharmaceuticals*)	IGF-1R, IR	Phase II	NCT01101906
Cixutumumab (IMC-A12; *ImClone Systems Inc*)	IGF-1R	Phase II	NCT00639509
BIIB022 (*Biogen-Idec*)	IGF-1R	Phase I	NCT00555724
AVE1642 (*Sanofi-Aventis*)	IGF-1R	Phase I/II	NCT00791544
Everolimus (RAD001; *Novartis*)	mTOR	Phase I/II	NCT00390195
Temsirolimus (Torisel; *Wyeth Pharmaceuticals, Inc*)	mTOR	Pahse II	NCT01079767; NCT01251458
AZD8055 (*AstraZeneca)*	mTOR	Phase I/II	NCT00999882
ARQ197 (*ArQule, Inc*)	c-Met	Phase I/II	NCT00802555; NCT00988741
MK-2206 (*Merck & Co., Inc.*)	Akt	Phase II	NCT01239355
AZD6244 (ARRY-142886, Selumetinib; *AstraZeneca*)	MEK	Phase I/II	NCT00550719; NCT00604721
**Combination of targeted agents**		**Design**	**Clinicaltrials.gov Identifier**
Sorafenib + Erlotinib		Phase III	NCT00901901
Sorafenib + AVE1642		Phase I/II	NCT00791544
Sorafenib + BIBF 1120			NCT01004003
Sorafenib + Panobinostat (LBH589, *Novartis*)		Phase I	NCT00823290
Sorafenib + Cixutumumab		Phase I	NCT01008566; NCT00906373
Sorafenib + OSI-906		Phase III	NCT01334710
Sorafenib + BIIB022		Phase I	NCT00956436
Sorafenib + Temsirolimus		Phase I/II	NCT01008917; NCT01335074
Sorafenib + ARQ197		Phase I	NCT00827177
Sorafenib + AZD6244		Phase I/II	NCT01029418
Erlotinib + Bevacizumab		Phase II	NCT01180959; NCT00242502; NCT00287222; NCT00365391
Erlotinib + AVE1642		Phase I/II	NCT00791544
Erlotinib + Celecoxib		Phase I/II	NCT00293436
Bevacizumab + Everolimus		Phase II	NCT00775073
**Targeted agents in combination with cytotoxic therapy**		**Design**	**Clinicaltrials.gov Identifier**
Erlotinib + Gemcitabine-Oxaliplatin (GEMOX)		Phase II	NCT00832637
Erlotinib + Docetaxel		Phase II	NCT00047333; NCT00532441
Cetuximab + Capecitabine-Oxaliplatin (CAPEOX)		Phase II	NCT00483405
Bevacizumab + transarterial chemoembolisation (TACE)		Phase II	NCT00280007
Bevacizumab + Gemcitabine-Oxaliplatin (GEMOX)		Phase II	NCT00142467

## PI3K/PTEN/AKT/MTOR PATHWAY

The PI3K/PTEN/Akt/mTOR pathway is another key pathway in HCC, its activation inducing cell proliferation and increasing survival. This pathway is activated after the binding of different growth factors to specific cell surface receptors, such as EGFR and IGF-1R (Figure 2). PI3K is a heterodimeric protein with an 85-kDa regulatory subunit and a 110-kDa catalytic subunit (*PIK3CA*). PI3K serves to phosphorylate a series of membrane phospholipids including PtdIns(4)P and PtdIns(4,5)P_2_, thereby forming the second messenger lipids PtdIns(3,4)P_2_ (PIP2) and PtdIns(3,4,5)P_3_ (PIP3). PIP3 then activates the phosphotidylinositide-dependent kinases (PDKs) which are responsible for activation of serine-threonine kinase Akt/protein kinase B (PKB) (Figure 2). Once activated, Akt leaves the cell membrane to phosphorylate intracellular substrates, including caspase-9 [65], the pro-apoptotic molecule BAD [66, 67], GSK-3β [68], and kinase IκB (IKK) [69]. When these targets are phosphorylated by Akt, they may either be activated or inactivated (*e.g.* phosphorylated BAD is inactive), but the final result is to promote cell survival. As well as intracellular substrates, Akt is able to target a number of transcription factors. In fact, after activation Akt is able to translocate into the nucleus [70] where it affects the activity of a number of transcriptional regulators, such as cAMP response element-binding (CREB) [71], E2F [72, 73], NF-κB (via IKK) [69], and the forkhead transcription factors [74-77].

Activated Akt positively modulates mTOR function. mTOR phosphorylates components of the protein synthesis machinery, such as the serine-threonine kinase p70^S6^ (40S ribosomal protein kinase) and the translation repressor eukaryotic initiation factor 4E-binding protein-1 (4E-BP1), both regulating the translation of important factors involved in cell proliferation (such as c-myc, cyclic D1 and pRb) and angiogenesis (such as HIF1-α).

Negative regulation of the PI3K pathway is primarily accomplished through the action of the PTEN tumor suppressor protein. PTEN in turn dephosphorylates PIP3, thus inhibiting the PI3K/Akt pathway.

Activation of PI3K/PTEN/Akt/mTOR signaling through the mutation, inactivation or silencing of pathway components occurs in various malignancies, including HCC [78]. Deregulation of this pathway has been documented to have clinical importance in HCC. For example, recent data from a genomic sequence of HCC samples identified mutations in *PIK3CA*, an upstream regulator of Akt, in 50% of patients with poor prognosis and survival length < 3 years following partial liver resection, whereas only 10% of the HCC patients with a good prognosis had a mutation in *PIK3CA* [79]. Activation of Akt is a risk factor for early disease recurrence and poor prognosis in patients with HCC [54, 80]. Several mechanisms may be responsible for the activation of Akt. The high frequency of *PIK3CA* mutations and/or its upregulation in patients with a shorter survival might be responsible for the Akt hyperactivation found in HCC with poor prognosis [80]. Selective epigenetic silencing of multiple inhibitors of the Ras pathway also seems to be responsible for the activation of Akt found in HCC [78]. Moreover, impaired expression of PTEN is involved in the regulation of Akt activity. Activation of Akt signaling and a reduced expression of PTEN has been reported in 40–60% of human HCC [80].

The best evidence strongly supporting the connection between PTEN suppression and liver carcinogenesis comes from genetic studies. All mice with PTEN-deficient hepatocytes exhibited liver adenomas and 66% of them developed HCC [81]. In these mice, hepatocytes were hyperproliferative and displayed an abnormal activation of Akt [81]. Furthermore, although mutations in the PTEN gene rarely occur in HCC, frequent loss of heterozygosity of the PTEN allele has been identified in 20–30% of HCC patients [82-85]. In addition, downregulation of PTEN expression may be partly due to PTEN promoter methylation [86]. Recent studies have also demonstrated that PTEN expression plays a critical role in HCC progression and patient survival. Patients with a high PTEN expression had a significantly better overall survival than patients with a low expression [87, 88].

An important role of the PI3K/PTEN/Akt/mTOR pathway has been suggested for HCC progression in obese patients. In the study by Saxena et al., leptin not only promoted HCC growth and invasiveness through activation of ERK pathway, but also through activation of PI3K/PTEN/Akt/mTOR signaling [55]. The other well-known risk factors, HBV and HCV, also seem to utilize the PI3K/PTEN/Akt/mTOR pathway to control hepatocyte survival and viral replication [89, 90]. It has been reported that HBx expression downregulated PTEN expression in hepatocytes [91]. In contrast, PTEN expression in liver cells downregulated HBx-induced PI3K and Akt activities [92]. Therefore, these studies suggest the possible use of PTEN as a target in therapeutic approaches, at least for the treatment of HCC caused by HBV infection.

Recent studies have demonstrated that mTOR inhibition shows a remarkable activity against a wide range of human cancers *in vitro* and human tumor xenograft models. The mTOR pathway is known to be upregulated in a subset of HCC patients [93]. In this study 15% of HCC displayed overexpression of phospho-mTOR, whereas 45% of HCC had increased expression of p70 S6K, which correlated with tumor nuclear grade. The importance of the mTOR pathway in HCC was confirmed by Llovet's group in a comprehensive study with 314 HCC and 37 non-tumor tissues using a series of molecular techniques to assess mutation, DNA copy number changes, messenger RNA and gene expression, as well as protein activation [94]. Aberrant activation of mTOR signaling (p-RPS6) was present in half of the cases and was associated with IGF pathway activation, EGF up-regulation, PTEN dysregulation and chromosomal gains in the rapamycin-insensitive companion of mTOR (RICTOR) (25% of patients). Furthermore, positive p-RPS6 staining correlated with HCC recurrence after resection [94]. Overall, these data support efforts to target mTOR signaling in liver cancer patients.

Taken together, these data suggest that the PI3K/PTEN/Akt/mTOR pathway may represent an important therapeutic target for HCC treatment in patients with differing etiologies that lead to the development of this aggressive tumor.

## IGFR PATHWAY

The IGF-I receptor (IGFR) signaling system consists of circulating ligands – IGF-I and IGF-II – interacting with a membrane receptor, such as type I IGF receptor (IGF-1R). The IGF-1R is a heterotetramer consisting of two extracellular ligand-binding α subunits and two β subunits with transmembrane and TK domains (Figure 1). Upon ligand binding IGF-1R undergoes conformational changes and phosphorylation, leading to the recruitment of insulin-receptor substrates (IRS) and/or Src homology 2 domain-containing (Shc) proteins, with the consequential activation of pathways also common to EGFR, including the PI3K/Akt/mTOR-axis and the Ras/MEK/ERK-pathway (Figure 2).

Constitutive activation of the IGF-signaling axis is frequently observed in a wide variety of tumors, including HCC [95, 96]. The overexpression of IGF-II, IGF-1R, and IRS contributes to cell proliferation and the inhibition of apoptosis, as well as increasing invasive behavior in HCC [97]. In HCC the reactivation of IGF-signaling predominantly occurs at the level of IGF-II expression [98, 99], but not of IGF-I. Overexpression of IGF-II has been observed in 16-40% of human HCC and around 30% of HCC cases overexpress IGF-1R [99, 100]. IGF-II overexpression is mainly due to altered methylation of the *IGF-2* gene promoters P1-P4 [101]. Furthermore, in HBV- and HCV-associated HCC, the HBV-derived HBx protein and HCV-derived core gene product have been reported to facilitate IGF-II overexpression [102, 103]. Moreover, in animal models of HCC the IGF signaling system also seems to be responsible for the development of HCC in obese and diabetic mice. Since obesity and diabetes are clearly associated with an increased risk of cancer in humans [104, 105], these observations highlighted the pivotal role of IGF signaling system in these patient categories.

## WNT/Β-CATENIN PATHWAY

The *Wnt* gene family encodes secreted glycoproteins involved in cell growth, differentiation, organogenesis, and oncogenesis. In a normal steady state (in the absence of Wnt proteins) β-catenin, the central player in the canonical Wnt pathway, is phosphorylated at amino-terminal serine and threonine residues by casein kinase 1 (CK1) and glycogen synthase kinase 3β (GSK-3β). β-catenin phosphorylation is facilitated by the scaffolding proteins axin and adenomatous polyposis coli (APC). Phosphorylated β-catenin is targeted for ubiquitination and protein degradation by the proteasome (Figure 3). Wnt signaling events are initiated by the binding of Wnt proteins to the seven-pass transmembrane Frizzled (FZD) receptor and the coreceptor low-density lipoprotein–related protein (LRP) 5/6. Then, Dishevelled (Dvl) is recruited to the FZD receptor, and the FZD/Dvl complex subsequently relocates axin to LRP5/6. The recruitment of axin to LRP5/6 is mediated by phosphorylation of LRP5/6 on key residues by the kinases CK1 and GSK-3β, which ultimately leads to GSK-3β inactivation. The absence of β-catenin phosphorylation releases it from the degradation complex composed of APC, axin, GSK-3β and CK1, resulting in an accumulation of β-catenin in the cytoplasm, since it cannot be degraded by the ubiquitin-proteasome pathway. As a consequence, β-catenin translocates into the nucleus where it binds to the lymphoid enhancer factor (LEF) or T-cell factor (TCF) transcriptional factors, displacing the transcriptional inhibitor Groucho, and in complex with LEF/TCF activates the expression of different genes which regulate cell proliferation and apoptosis (Figure 3).

A role for Wnt/β-catenin signaling in HCC was discovered over a decade ago [106]. Activating mutations in the β-catenin gene (CTNNB1) were found in different human HCC cell lines and in HCC clinical samples in around 20%-40% of all cases [106-112]. These mutations impair the GSK-3β-mediated phosphorylation of the protein at serine and threonine residues in its N-terminus region. Intriguingly, HCC occurring in HCV patients showed a high incidence of β-catenin gene mutations (up to 40% of cases) [109, 113], whereas in HCC occurring in HBV patients β-catenin activation is induced in a mutation-independent manner by the expression of HBx protein [114, 115]. However, in the absence of β-catenin gene mutations, aberrant activation of β-catenin has been identified in a significant subset of HCC patients with mutations in axin1/2 (5%) [116]. The observation that expression of the wild-type *AXIN1* gene by adenovirus mediated gene transfer induced apoptosis in HCC cells, which had accumulated β-catenin as a consequence of either *APC*, *CTNNB1* or *AXIN1* gene mutation, highlights the fact that axin may be an effective therapeutic molecule for suppressing HCC growth [116]. Recently, since axin is the concentration-limiting component of the β-catenin destruction complex, stabilization of axin by inhibiting the poly-ADP-ribosylating enzymes tankyrase 1 and tankyrase 2 with small molecule inhibitor XAV939 has been presented as a new avenue for targeted Wnt/β-catenin pathway therapies [117].

Moreover, accumulation of β-catenin in human HCC tumors containing the wild-type β*-*catenin gene has been observed in the context of up-regulation of the FZD7 receptor, which has been found up-regulated in 90% of human HCC [118-120], suggesting that *FZD7* gene expression is the most common abnormality observed in HCC and consequently activation of Wnt/Frizzled-mediated signaling plays a key role in liver carcinogenesis. Accordingly, Nambotin et al. demonstrated that pharmacological inhibition of FZD7 displayed anti-cancerous properties against HCC *in vitro* (on a panel of human HCC cell lines) and *in vivo* (on the SV40–TAg transgenic mouse model of HCC) [121]. Therefore, these observations suggest that the Wnt/β-catenin signal transduction pathway is much more commonly involved in the molecular pathogenesis of HCC than previously recognized. Although no clinical studies are available, a preclinical study in which β-catenin suppression was achieved by antisense modalities has shown that β-catenin is essential for the survival and growth of hepatoma cells, independently of mutations in the β-catenin gene, and therefore this provides a proof of principle for the significance of the therapeutic inhibition of β-catenin in HCC [122].

## HEDGEHOG PATHWAY

The Hedgehog (Hh) pathway is essential for embryonic development, tissue polarity and cell differentiation [123-125]. This pathway is critical in the early development of the liver and contributes to differentiation between hepatic and pancreatic tissue formation [126]. It remains inactive in healthy adult liver tissue, except during tissue regeneration and remodeling tissue repair, and Hh signaling may also play a role in primary liver cancers, such as cholangiocarcinoma and HCC [127, 128]. The Hh signaling pathway is complex and requires two cellular receptors, Patched-1 (Ptch-1) receptor and Smoothened (Smo), a 7-transmembranous domains protein receptor. In the absence of ligand, Ptch-1 represses Smo, thereby silencing the Hh signaling pathway. Binding of the Hh ligands - Sonic Hedgehog (Shh), Indian Hedgehog (Ihh) and Desert Hedgehog (Dhh) - to Ptch-1 liberates Smo from Ptch-1-mediated inhibition, thus initiating the propagation of an intracellular signaling cascade that leads to the activation and nuclear translocation of glioma-associated oncogene homologue (Gli) family transcription factors (Gli1, Gli2, Gli3) which regulate the expression of Gli-target genes [129]. The different Gli proteins show activating or repressing transcriptional activators depending on proteolytic cleavage of the full-length proteins. Gli1 and Gli2 mainly act as transcriptional activators, whereas in the absence or inhibition of Hh signaling processing of Gli3 produces a repressor form (Gli3R).

Hh has emerged as a critical mediator in the development of various diseases, including cancer, when aberrantly activated [130].

Although the study of Hh signaling in liver cells is in its infancy, some studies have shown that activation of the Hh pathway is involved in liver carcinogenesis [131-136]. Therefore, blockade of the Hh signaling pathway may be a potential new therapeutic strategy in HCC.

The relevance of blocking the Hh pathway for HCC treatment can be further supported by the evidence that this pathway can cross-talk with the Wnt/β-catenin signaling pathway, a well-known oncogenic pathway implicated in HCC development [137, 138]. Taken together, these data suggest that inhibition of the Hh pathway may provide a useful therapeutic option for the treatment of HCC.

## INFLAMMATORY PATHWAY (IL-6/STAT3, TNF-α, NF-κB, COX-2)

The link between inflammation and cancer was first suggested by Rudolph Virchow in 1863, and is now a widely-accepted paradigm of carcinogenesis [139, 140]. Nowadays epidemiological data have undoubtedly demonstrated a clear association between chronic inflammation and tumor development, including HCC [141-143]. Although the molecular mechanisms by which chronic inflammation increases the risk of HCC are not completely known, compelling evidence gathered over the past few years has demonstrated the roles of inflammatory factors, such as IL-6, cyclooxygenase 2 (COX-2)/prostaglandin E_2_ (PGE_2_) and tumor necrosis factor α (TNF-α) in HCC development [142].

IL-6 mediates its diverse biological effects by interacting with a receptor complex consisting of a specific ligand-binding protein (IL-6R, gp80) and a signal transduction protein (gp130) and regulates the JAK/STAT3, Ras/MAP kinase and PI3K/Akt pathways. A key feature in our understanding of the regulation of IL-6 responses has been the identification of a soluble form of the IL-6 receptor (sIL-6R) [144]. When the IL-6/sIL-6R complex associates with the membrane-bound signal-transducing chain, it can induce the signal transduction cascade, acting as an agonist and stimulating a variety of cellular responses including the proliferation, differentiation and activation of inflammatory processes.

A large body of evidence has been accumulating in recent years which indicates that IL-6 is involved in liver carcinogenesis [143, 145]. In this line, Michael Karin's group showed that IL-6 participates in hepatocarcinogenesis, using diethylnitrosamine (DEN)-induced murine HCC models [146]. They also showed that estrogen-mediated inhibition of IL-6 production by Kupffer cells reduces liver cancer risk in females and these findings not only may be used to prevent HCC in males, but also may be a possible clue for the enigma of gender difference in HCC occurrence found in epidemiologic data [147]. Recently, a retrospective cohort study was conducted to examine whether the results observed in the mouse models were applicable to human HCC [148]. No significant difference in serum IL-6 levels was found between female and male chronic hepatitis C patients. Unexpectedly, in a multivariate analysis higher serum IL-6 level was an independent risk factor for HCC development in female but not in male chronic hepatitis C patients. Therefore, the gender disparity in liver carcinogenesis in humans cannot be attributed solely to the difference in IL-6 levels. Interestingly, a recent report suggested that Foxa factors (Foxa 1 and Foxa2) and their targets are central for the sexual dimorphism of HCC [149]. The mechanism of gender disparity remains to be further investigated.

Nevertheless, many works have reported high serum levels of IL-6 in various liver diseases, including HCC. Serum IL-6 levels are significantly higher in patients with HCC than in healthy individuals [150-152] and higher levels of IL-6 have been correlated with tumor mass and cancer invasiveness [150, 153]. Moreover, IL-6 is much higher in stage III HCC patients than in stage I and II patients [151]. As regards sIL-6R, although no significant difference in sIL-6R levels were observed between control subjects and patients with HCC, sIL-6R levels resulted higher in patients with a more advanced stage of disease [151, 154].

STAT3 is the major mediator of IL-6 and growth factor (*e.g.* EGF, PDGF and HGF) signaling, transmitting signals from the cell membrane to the nucleus. STAT3 activation requires phosphorylation of a critical tyrosine residue (Tyr705), which mediates its dimerization, which is a prerequisite for nucleus entry and DNA binding. The phosphorylation of STAT3 at Tyr705 is most commonly mediated by Janus kinases (JAKs), especially JAK2. Activated STAT3 can mediate oncogenic transformation in cultured cells and promote tumor formation in nude mice, thus qualifying *STAT3* as a proto-oncogene [155]. STAT3 is constitutively activated in human HCC tissues, but not in adjacent non-tumor liver parenchyma or normal liver tissue [156, 157]. A recent report demonstrated that the STAT3 signaling pathway is very complex and may participate in HCC genesis and development by regulating the protein expression of other signaling pathways, telomerase, apoptosis, the cell cycle and angiogenesis [158]. Targeting STAT3 as a potential cancer therapy has been extensively investigated [159], and recently new small-molecule inhibitors have been developed which show to inhibit IL-6-induced STAT3 activation and nuclear translocation in HCC cells [160]. Therefore, targeting IL-6/STAT3 seems to be a promising strategy for HCC therapy.

An inducible enzyme with carcinogenic properties that is active within inflamed and malignant tissues is cyclooxygenase-2 (COX-2). The COX enzymes (COX-1 and COX-2) are well-known targets of non-steroidal anti-inflammatory drugs (NSAIDs). Many epidemiological studies have demonstrated that treatment with NSAIDs reduces the incidence and mortality of certain malignancies, especially gastrointestinal cancer [161]. However, conventional NSAIDs non-selectively inhibit both the constitutive form COX-1, and the inducible form COX-2. Recent evidence indicates that COX-2 is an important molecular target for anticancer therapies. Its expression is undetectable in most normal tissues, and is highly induced by pro-inflammatory cytokines, mitogens, tumor promoters and growth factors. It is now well-established that COX-2 is chronically overexpressed in many premalignant, malignant, and metastatic cancers [162], including HCC [163-165]. Overexpression of COX-2 in patients with HCC is generally higher in well-differentiated HCCs compared with less-differentiated HCCs or histologically normal liver, suggesting that COX-2 may be involved in the early stages of liver carcinogenesis [163-165] and increased expression of COX-2 in noncancerous liver tissue has been significantly associated with postoperative recurrence and shorter disease-free survival in patients with HCC [166, 167]. In tumors, overexpression of COX-2 leads to an increase in prostaglandin levels, which affect many mechanisms involved in carcinogenesis, such as angiogenesis, inhibition of apoptosis, stimulation of cell growth as well as the invasiveness and metastatic potential of tumor cells [168].

The availability of novel agents that selectively inhibit COX-2 (COXIB) has contributed to shed light on the role of this molecule. Experimental studies on animal models of HCC have shown that NSAIDs, including both selective and non-selective COX-2 inhibitors, exert chemopreventive as well as therapeutic effects [169-172]. However, the key mechanism by which COX-2 inhibitors affect HCC cell growth is as yet not fully understood. Increasing evidence suggests the involvement of molecular targets other than COX-2 in the anti-proliferative effects of COX-2 selective inhibitors, including the MAPK cascade [173, 174], PI3K/Akt pathway [175] and its upstream kinase PDK-1 [176], the anti-apoptotic proteins survivin, Bcl-2 and Mcl-1 [177, 178], cyclin-dependent kinase inhibitors and cyclins [179], as well as the sacroplasmic/endoplasmic reticulum calcium ATPase SERCA [180]. Interestingly, COX-2-independent effects of celecoxib have also been observed during liver carcinogenesis *in vivo.* In the study by Marquez-Rosado [169] neither COX-2 expression nor PGE_2_ production were altered by celecoxib treatment, suggesting that celecoxib effects are mediated by COX-2/PGE_2_-independent mechanisms.

Therefore, COX-inhibitors may use both COX-2-dependent and COX-2-independent mechanisms to mediate their antitumor properties [174, 181, 182], although their relative contributions toward the *in vivo* effects remain less clear.

Interestingly, celecoxib also inhibits IL-6/IL-6 receptor-induced JAK2/STAT3 phosphorylation in human HCC cells [183].

The NF-κB pathway has also been recognized as an underlying link between inflammation and malignancy [184]. The transcription factor NF-κB is a ubiquitous transcription factor present in all cell types. In unstimulated cells, NF-κB resides in the cytoplasm as a heterotrimer consisting of p50, p65, and IκBα. The binding of a ligand, such as cytokines or lipopolysaccharide (LPS), to a receptor leads to the recruitment and activation of an IκB kinase (IKK) complex, which consists of IKKα and/or IKKβ catalytic subunits and two molecules of NEMO. Phosphorylation of serine residues of IκB by IKK leads to IκB ubiquitination and subsequent proteosomal degradation. p50 and p65 are then released and translocated into the nucleus, where gene expression is activated. Most genes linked with tumorigenesis are regulated by NF-κB, such as those mediating inflammation, cell survival, cell proliferation, invasion, angiogenesis, and metastasis.

In recent years, several results have established strong support for the critical role of NF-κB in many types of cancer, including HCC [185, 186]. NF-κB is aberrantly expressed and activated in both human HCC tissue and HCC cells [187-189]. Several preclinical studies have shown that inhibition of NF-κB signaling by pharmacological or genetic approaches [189-192] results in an antitumor effect in HCC, suggesting that NF-κB is a potential molecular target for HCC therapy. Worthy of note is the observation that celecoxib potently inhibits the nuclear translocation and activation of NF-κB by COX-2-dependent and -independent mechanisms [169, 181, 193, 194]. Interestingly, we recently reported that combination of celecoxib with the novel NF-κB inhibitor dehydroxymethyl-epoxyquinomicin (DHMEQ) synergistically inhibits cell growth, NF-κB p65 DNA-binding capacity, and cell proliferation in human HCC cells [195], providing a rational basis for the clinical use of this combination in the treatment of liver cancer.

The important role of inflammatory pathways in liver carcinogenesis is further reinforced by recent studies by Michael Karin's team, published in *Cell* in 2010 [196]. Park et al. demonstrated that either dietary or genetic obesity is a potent *bona fide* liver tumor promoter in mice. Obesity-promoted HCC development was dependent on the production of the tumor-promoting cytokines IL-6 and TNF-α, which cause hepatic inflammation and activation of the oncogenic transcription factor STAT3. The chronic inflammatory response caused by obesity and enhanced production of IL-6 and TNF-α ma also increase the risk not only of HCC but of other cancers [105, 196].

## OTHER POTENTIAL THERAPEUTIC TARGETS IN HCC

As stated above, during the multistep biological process involved in the development of HCC several genetic and epigenetic alterations occur and various pathways are involved, including transforming growth factor (TGF)-β [197], hepatocyte growth factor (HGF)/c-MET [198-200], Hyppo [201] and Notch [202, 203] signaling. These molecules may represent critical therapeutic targets for HCC intervention as well as for other cancers.

## MOLECULAR-TARGETED THERAPY IN HCC

Several recent reviews have been published describing in detail the results of clinical trials of molecular-targeted agents for the treatment of HCC [204-208]. Here, we briefly review only some of them, whereas an updated list of data accessed up to February 2012 by searching the clinicaltrials.gov website on ongoing clinical trials in HCC patients is reported (Table 1).

## TARGETING THE RAF/MEK/ERK PATHWAY

The Raf kinase inhibitor sorafenib (Nexavar, BAY43-9006) is currently the most promising molecular targeting drug for HCC. Sorafenib, is a multikinase inhibitor, which in addition to targeting Raf kinases also inhibits VEGFR-2/-3, PDGFR-β, Flt-3 and c-Kit [42, 209, 210] (Figure 1). On the basis of the recent large randomized phase III study, the Sorafenib HCC Assessment Randomized Protocol (SHARP), Sorafenib has been approved by the United States (US) Food and Drug Administration (FDA) for the treatment of patients with advanced HCC [211]. In the SHARP trial median overall survival (OS) increased from 7.9 months in the placebo group to 10.7 months in the sorafenib group. Sorafenib showed a significant benefit also in terms of time to progression (TTP), with a median of 5.5 months in the sorafenib group and 2.8 months in the placebo group. On the basis of these findings, the FDA, European Medicine Agency (EMA) and other regulatory authorities in the world have approved sorafenib for advanced HCC treatment. However, although sorafenib is well tolerated, concern for its safety has been expressed. Most common adverse events reported in the SHARP trial were diarrhea and hand-foot skin reactions [211]. Sorafenib is currently undergoing investigation in a phase III study - the STORM trial - in HCC patients as an adjuvant therapy for the prevention of recurrence following surgery or local ablation (http://clinicaltrials.gov/ct2/show/NCT00692770).

In addition to sorafenib other molecular targeting agents have been used in clinical trials for advanced HCC treatment (Table 1). However, most of them have demonstrated very low responses. The low response rate associated with monotherapy indicates the need to explore combinations of different molecular targeting agents, but also combinations of a single agent with conventional cytotoxic drugs. In this context, a phase II trial demonstrated that the addition of sorafenib to doxorubicin improves progression-free and overall survival of patients with advanced HCC [212]. Moreover, a phase II trial is currently recruiting patients to determine the progression-free survival of sorafenib plus tegafur/uracil (UFUR) for the treatment of advanced or metastatic HCC (NCT00464919).

In addition to Raf inhibition, preclinical studies have demonstrated the potential of MEK inhibition to suppress hepatoma cell proliferation and tumorigenicity [44, 46, 213-216]. Huynh et al. recently reported that treatment of human HCC xenografts with AZD6244 (ARRY-142886, Selumetinib), a selective MEK inhibitor, blocked ERK1/2 activation, reduced *in vivo* tumor growth and induced apoptosis [44]. Targeting MEK with the selective MEK inhibitor PD0325901, a derivative of CI-1040, had *in vivo* chemopreventive effects on HCC development in an animal model employing TGF-α-transgenic mice with liver cancers induced by diethylnitrosamine treatment [217]. In addition, a combination of the MEK inhibitor AZD6244 and the conventional cytostatic drug doxorubicin enhanced the antineoplastic activity of the respective monotherapeutic HCC treatment with doxorubicin alone [218]. MEK inhibitors have also been shown to potentiate the antitumor activity of selective COX-1 and COX-2 inhibitors in suppressing growth and inducing apoptosis in human liver cancer cells [174].

Taken together, the *in vitro* and preclinical *in vivo* data show that MEK inhibitors are promising agents for HCC treatment. However, a multicenter phase II clinical study failed to demonstrate a clinical benefit for AZD6244 as a single agent in patients with advanced HCC [219]. This result suggests that inhibition of MEK signaling alone is not sufficient to successfully treat advanced-stage HCC, therefore two clinical trials are currently testing AZD6244 in HCC patients with less severe disease, i.e. moderate liver dysfunction, and also in association with sorafenib (Table 1).

## TARGETING THE PI3K/AKT/MTOR PATHWAY

The PI3K/Akt/mTOR pathway appears to be one of the major contributors to the development and maintenance of HCC. Although some preclinical studies have demonstrated that PI3K inhibitors such as perifosine, LY29004 and wortmannin have anti-HCC activity, no studies have been conducted so far at the clinical level.

A phase II Study of MK-2206 (a novel oral potent allosteric Akt inhibitor) in advanced HCC patients who have not responded or are intolerant to one previous line of anti-angiogenic therapy is currently recruiting patients (NCT01239355). Of interest, a recent study showed that the combination of sorafenib and MK-2206 overcomes the resistance of HCC cells to sorafenib at clinically achievable concentrations, suggesting the potential use of this treatment in HCC patients [220].

Evidence from *in vitro* experiments, as well as from preclinical *in vivo* data, indicated that mTOR inhibition by rapamycin and its analogues everolimus (RAD001) significantly reduced the growth of HCC cells and improved survival primarily via antiangiogenic effects [221-224].

A pilot study conducted on 21 patients with advanced HCC indicated that sirolimus (rapamycin) was a promising drug for the treatment of HCC and a randomized phase I/II trial evaluating the rapamycin analog RAD001 (everolimus) for advanced HCC is currently recruiting patients (http://clinicaltrials.gov/ct2/show/NCT00516165).

Other clinical trials are ongoing to evaluate dose limited toxicity and efficacy in advanced HCC patients treated with the mTOR inhibitor Torisel (temsirolimus). Furthermore, a phase I/II multicentre study to assess the safety, tolerability, pharmacokinetics and preliminary efficacy of AZD8055, a novel ATP-competitive inhibitor of mTOR kinase, is recruiting Asian patients with advanced stage HCC (Table 1).

A topic of considerable current interest concerns the signal transduction pathways and molecular mechanisms linked to the chemoresistance of tumor cells to conventional anticancer drugs. In this context, a combination of rapamycin with the conventional cytostatic drugs doxorubicin and vinblastine enhances the antineoplastic activity of the respective monotherapeutic HCC treatment with either doxorubicin or vinblastine alone [225, 226].

In addition to studies on the combination of mTOR inhibitors with conventional chemotherapeutic agents, two phase I/II clinical studies are currently recruiting patients with advanced HCC to determine the safety/toxicity profile of temsirolimus in combination with sorafenib (Table 1).

Taken together, the *in vitro* and preclinical *in vivo* data, as well as the clinical trials, conducted so far demonstrate that mTOR inhibitors are promising agents for HCC treatment, particularly in combination with conventional chemotherapeutic drug therapy.

## TARGETING THE VEGF/VEGFR, FGF/FGFR AND PDGF/PDGFR PATHWAYS

HCC is a hypervascular tumor mainly supplied by the hepatic arteries and secretion by HCC cells, tumor-infiltrating inflammatory cells and hepatic stellate cells of factors such as VEGF, bFGF, angiopoietins, PDGF and others promotes the sprouting of new vessels from nearby existing vessels.

VEGF, is one of the strongest stimulatory angiogenic factors, and is up-regulated in most human tumors, including HCC [227, 228]. In a recent systemic review and meta-analysis study, the prognostic role of VEGF as a predictor of survival in patients with treated HCC was established [229]. High tissue VEGF levels predicted poor overall (HR=2.15, 95% CI: 1.26-3.68) and disease-free (HR=1.69, 95% CI: 1.23-2.33) survival. Similarly, high serum VEGF levels predicted poor overall (HR=2.35, 95% CI: 1.80-3.07) and disease-free (HR=2.36, 95% CI 1.76-3.16) survival. Therefore, the inhibition of angiogenesis may represent a potential therapeutic target in HCC, and many antiangiogenic agents are under evaluation in clinical trials in HCC.

Bevacizumab is a recombinant humanized monoclonal antibody against VEGF which has been used either as a single agent or in combination with cytotoxic or other targeted agents in several clinical studies already concluded in patients with advanced HCC [230-236], whereas others are still recruiting patients (Table 1). Overall, the concluded studies demonstrated that although bevacizumab is a well-tolerated agent, the side effects associated with its administration, including bleeding, hypertension, proteinuria, and thromboembolic events, warrant further evaluation.

Other multiple RTK inhibitors that target VEGF are under investigation, including brivanib, linifanib (formerly ABT-869), vandetanib, and pazopanib.

Recently, in a phase II trial brivanib, a selective dual inhibitor of VEGF and FGF signaling, was evaluated as a first-line therapy in patients with unresectable, locally advanced or metastatic hepatocellular carcinoma. The study showed a median OS of 10 months. Brivanib was generally well tolerated; the most common adverse effects included fatigue, hypertension, and diarrhea [237]. Based on these results a randomized, double-blind, multi-center phase III study of brivanib versus sorafenib as first-line treatment is currently testing the OS of patients with advanced HCC who have not received prior systemic therapy (NCT00858871), whereas another phase III trial, the BRISK PS Study (Brivanib Study in HCC Patients at Risk Post Sorafenib), is evaluating brivanib plus best supportive care (BSC) versus placebo plus BSC in subjects with advanced HCC who have not responded or are intolerant to sorafenib (NCT00825955).

Linifanib (ABT-869) is a novel orally active, potent and selective inhibitor of the VEGF and PDGF receptor tyrosine kinases. A phase II study on 44 patients with advanced HCC showed a response rate of 7%, a median PFS of 3.7 months and median survival of 9.3 months [238]. This study concluded that linifanib is clinically active in advanced HCC, with an acceptable safety profile. On the basis of these results, a phase III study of linifanib versus sorafenib is ongoing.

A phase II, placebo-controlled study of vandetanib (ZD6474), which targets VEGFR, EGFR and RET signaling, showed activity in patients with inoperable HCC but failed to meet its primary aim of tumor stabilization [239]. However, the PFS and OS results suggest that vandetanib has clinical activity in this patient population that may warrant further investigation.

Finally, a report from a phase I dose-ranging study of pazopanib (GW786034), an oral inhibitor targeting VEGF, PDGF and c-kit, showed evidence of antitumor activity [240].

## TARGETING THE EGFR PATHWAY

Another promising target in HCC is the EGFR pathway. As mentioned above, EGFR and its ligand EGF play an important role in hepatocarcinogenesis. Two therapeutic approaches are currently being employed in clinical trials in HCC patients, by using either a monoclonal antibody neutralizing the EGFR (cetuximab) or three small-molecule tyrosine kinase inhibitors of the EGFR (erlotinib, gefinitib and lapatinb). Overall, the results have been disappointing. Indeed, in phase II clinical trials in which erlotinib, gefitinib, lapatinib and cetuximab were assessed in patients with advanced HCC response rates (RR) varied in the range of 0%–9%, the median PFS time reported was approximately 1.4–3.2 months and OS ranged 6.2-13 months [241-244]. Consequently, several ongoing clinical trials are combining EGFR inhibitors with another therapeutic modality such as cytotoxic drugs and other molecular-targeted agents [235, 236, 245, 246] (Table 1).

## TARGETING THE IGF PATHWAY

Constitutive activation of the IGF-signaling axis is frequently observed in HCC [95,96]. In HCC the activation of IGF-signaling has antiapoptotic and growth-promoting effects and acts through multiple signaling cascades, including the PI3K/Akt and MAPK pathways. As for other pathways, small molecules (such as OSI-906) and monoclonal antibodies (such as IMC-A12 and AVE-1642) targeting IGF signaling are under evaluation in clinical trials in HCC patients (Figure 1 and Table 1).

Pre-clinical evidence obtained *in vitro* in HCC cells showed that IMC-A12 decreased cell viability and proliferation and blocked ligand-induced IGF-1R activation. *In vivo* A12 delayed tumor growth and prolonged survival, reducing proliferation rates and inducing apoptosis [247]. Therefore, these data suggest that IMC-A12 effectively blocks IGF signaling, thus providing the rationale for testing this therapy in clinical trials. Indeed, an initial phase I study of IMC-A12 (cituxumumab) yielded a partial response in HCC [248], however a subsequent phase II study in patients with advanced HCC showed that IMC-A12 is inactive as a monotherapy in HCC [249].

AVE1642 is a humanized monoclonal antibody that specifically blocks IGF-1R signaling. A phase I study showed that AVE1642 can be safely combined with active doses of sorafenib, and the pharmacokinetics of both AVE1642 and sorafenib were not modified at the concentrations tested. Interestingly, long-lasting disease stabilizations were observed in most patients with progressive disease [250].

Recently, OSI-906, a novel orally-efficacious small-molecule dual IGF-1R/Insulin receptor (IR) kinase inhibitor has been isolated and is being evaluated as a therapeutic agent for HCC [251]. OSI-906 is currently being tested in a randomized, placebo-controlled, double-blinded phase 2 study of second-line treatment in patients with advanced HCC after failure of first-line treatment with sorafenib (NCT01101906; Table 1).

## CONCLUSIONS

The recent identification of several key molecular pathways implicated in the pathogenesis of HCC has led to the development of new targeted therapies for this devastating disease. Targeting the various effectors of these pathways with pharmacologic inhibitors may inhibit HCC cell growth and angiogenesis. Several promising novel anticancer agents are currently under investigation for the treatment of HCC. Ongoing clinical trials are offering hope to improve the progression-free survival of patients with advanced HCC.

The specific action of the new molecular-targeted agents minimizes the toxicity typical of systemic chemotherapy, although attention needs to be paid to the onset and management of side effects related to treatment with these new agents.

Combination therapy with either conventional cytotoxic drugs or another inhibitor which targets a specific molecule in a different signal transduction pathway is also a key approach for improving the effectiveness and usefulness of new molecular-targeted agents. This avenue of investigation has not been pursued as rigorously as it could be, often due to the conflicting interests of the pharmaceutical companies, since different companies will often have competing interests for the different inhibitors/chemotherapeutic drugs. Nevertheless, the field of molecular-targeted therapy in cancer therapy has already come a long way. It is not hard to see an even brighter future on the horizon. However, many additional clinical trials, as well as the development of novel, innovative approaches to cure or suppress the further development of HCC need to be performed and developed to improve therapy in HCC patients.
